# Development SPE-LC-MS/MS method for determination of WHO AWaRe Reserve antibiotics in hospital wastewater

**DOI:** 10.1038/s41598-025-04951-z

**Published:** 2025-07-01

**Authors:** Joanna Wilk, Paulina Sowik, Ewa Felis, Monika Harnisz, Ewa Korzeniewska, Sylwia Bajkacz

**Affiliations:** 1 Department of Inorganic Chemistry, Analytical Chemistry, and Electrochemistry, Faculty of Chemistry, Silesian University of Technology, Krzywoustego 6 Str., 44-100 Gliwice, Poland; 2 Department of Environmental Biotechnology, Faculty of Energy and Environmental Engineering, Silesian University of Technology, Akademicka 2 Str., 44-100 Gliwice, Poland; 3 Department of Engineering of Water Protection and Environmental Microbiology, Faculty of Geoengineering, University of Warmia and Mazury in Olsztyn, Prawocheńskiego 1 Str., 10-720 Olsztyn, Poland; 4 Biotechnology Centre, Silesian University of Technology, Krzywoustego 8 Str., 44-100 Gliwice, Poland

**Keywords:** WHO AWaRe, Reserve antibiotics, Last-resort antibiotics, SPE-LC-MS/MS, Hospital wastewater, Environmental pollution, Environmental sciences, Chemistry

## Abstract

Overuse and inadequate legal regulation of the use of antibiotics have led to antimicrobial resistance, which threatens healthcare by reducing the effectiveness of treatments and promoting multidrug-resistant “superbugs”. The World Health Organization created the AWaRe (Access, Watch, Reserve) classification to categorize antimicrobials according to their therapeutic value, emphasizing prudent use. The most important group in terms of protecting effectiveness is the Reserve group. The aim of the study was to develop a method for the determination of the following Reserve antibiotics in hospital wastewater: aztreonam, linezolid, meropenem, tigecycline, vaborbactam, cilastatin, fosfomycin, and ceftazidime. Twenty-six extraction procedures were tested and the one with the best recoveries for all antimicrobials (31.5–103.7%) was selected. The extracts were analyzed by liquid chromatography-tandem mass spectrometry. The method was validated and applied to the determination of the selected pharmaceuticals in effluents from 16 hospitals in Poland, with Reserve antibiotics detected in each sample at concentrations ranging from 1.89 ng L^− 1^ to 22.49 µg L^− 1^. This is the first country-scale methodological study on Reserve antibiotics in hospital wastewater, emphasizing the need for early environmental monitoring and the potential integration of such strategies into healthcare systems to mitigate the global antimicrobial resistance crisis.

**Supplementary Information:**

The online version contains supplementary material available at 10.1038/s41598-025-04951-z.

## Introduction

Antimicrobial resistance (AMR) is a phenomenon that leads microorganisms to develop insensitivity to a drug that would, under normal conditions, cause their death or growth inhibition^1^. Overuse and improper disposal of antimicrobials (AMs) have contributed to the increased scale of this problem due to environmental pollution^2–4^.  A significant public health concern is the ability of bacteria to become insensitive to multiple AMs simultaneously, known as multidrug resistance (MDR). Treatment in such cases becomes particularly challenging and expensive due to the limited number of effective AMs^5^. It was estimated that 4.95 million deaths in 2019 were related to AMR^1^. This, combined with the difficulty of obtaining new compounds with antibacterial properties^6^, indicates the necessity to preserve the efficacy of currently available AMs. In response to this problem, the World Health Organization (WHO) created the AWaRe Classification, which categorizes antimicrobial agents into three groups: Access, Watch, and Reserve. The division is based on the risk of developing resistance and emphasizes the prudent use of AMs. The Access group includes first choice drugs for treating common infections. AMs from the Watch group have a higher risk of resistance development, so their use should be monitored and limited. The third group includes last-resort Reserve antimicrobials (RAMs) intended for use against diseases caused by MDR strains when other treatments have failed. Regulating their intake and monitoring their fate after administration is critical to preserving them for future use and should be considered a priority for antibiotic stewardship programs in every country^7–9^. As previously stated, environmental pollution with AMs profoundly impacts the development of drug resistance among microorganisms. The most probable initial point of entry of last-chance antibiotic residues and resistance genes into the environment is wastewater from hospitals^10^, where the most severe cases of infections with MDR bacteria strains are treated. In some countries, hospitals lack onsite wastewater treatment plants (WWTPs), and effluents from these facilities are combined with municipal wastewater. Moreover, it has been reported that in developing countries, hospital wastewater is discharged directly into surface water without prior pretreatment^11,12^. WWTPs employing conventional treatment methods cannot completely remove pharmaceuticals, so they continue to enter the environment. The majority of AMs enter biological treatment systems, where they influence microbial activity in the activated sludge. More advanced methods capable of removing AMs mostly involve the construction of expensive and complicated equipment with constricted application^13,14^. These substances are dispersed through various pathways, beginning with surface water. They can persist in the water or enter the sediment and river biofilm, depending on the physical and chemical properties of the compound and the matrix^15^. Further, they can migrate into groundwater and soil, where they can affect the microorganisms present there^16,17^. AMR can develop even in the presence of very low concentrations of AMs, as low as pg mL^−1^which continues to pose a public health risk and is an additional indication to reduce the environmental burden of antibiotics^18^. In their review, Wilk and Bajkacz compiled methods for determining RAMs in environmental samples. Notably, the review revealed a lack of published studies and of methods that allow the simultaneous determination of multiple drugs in this group. The primary technique employed in the described studies was a combination of solid-phase extraction (SPE) and liquid chromatography coupled with tandem mass spectrometry (LC-MS/MS).^19^ Meropenem was one of the most frequently detected last-chance AMs in hospital wastewater^20–22^, but also in local WWTPs influent and effluent^23–25^, where it must have entered directly from hospital outflows. Another frequently reported RAM was linezolid, which was even found in surface water in Bangladesh^26^. Studies conducted in the United States showed the presence of this RAM in WWTP influent (up to 61.5 ng mL^− 1^) and effluent (up to 22.1 ng mL^− 1^), indicating that it wasn’t removed during treatment^27^. The fact that the studies described in the review were conducted across five continents indicates that the problem of environmental contamination by RAMs is a global phenomenon^19^. This highlights the necessity to control last-resort AMs at environmental entry points, such as hospital wastewater outlets. The aim of this work was to develop an efficient and simple method for the determination of RAMs, including fosfomycin, tigecycline, linezolid, meropenem, vaborbactam, aztreonam, ceftazidime, and cilastatin, in wastewater collected from Polish public hospitals. The study included the development of an SPE procedure and determination by LC-MS/MS. Prior to the assessment of environmental samples, the obtained method was validated. The results of the analyses for hospital wastewater from each voivodeship (the highest administrative unit in Poland) are presented to show the prevalence of the selected RAMs across Poland.

## Materials and methods

### Chemicals and materials

The vaborbactam (VBR) reference standard was obtained from Cayman Chemical Company (Ann Arbor, MI, USA). Standards for the remaining RAMs, including fosfomycin (FOF), tigecycline (TGC), linezolid (LZD), meropenem (MEM), aztreonam (ATM), ceftazidime (CFD), and cilastatin (CIL), were sourced from MilliporeSigma (Burlington, MA, USA). LC-MS grade reagents such as acetonitrile (ACN), methanol (MeOH), water, ammonium acetate (≥ 99%), and formic acid (FA) (≥ 99%) were purchased from Avantor (Radnor, PA, USA). Additionally, pure MeOH, hydrochloric acid (35–38%), acetone, and ammonia (25%) were acquired from Chempur (Ruda Śląska, Poland). Solid ethylenediaminetetraacetic acid disodium salt (EDTA) and 4-morpholinepropanesulfonic acid (MOPS) were procured from MilliporeSigma. High-purity nitrogen (5.0 grade) was supplied by SIAD (Ruda Śląska, Poland).

ZORBAX Eclipse Plus C18 (4.6 × 50 mm, 5 μm), ZORBAX RRHD Eclipse Plus C18 (2.1 × 50 mm, 1.8 μm), and Poroshell 120 EC-C18 (3.0 × 50 mm, 2.7 μm) chromatographic columns were sourced from Agilent Technologies (Santa Clara, CA, USA). The Kinetex C18 (2.1 × 50 mm, 2.6 μm) and Kinetex F5 (2.1 × 100 mm, 1.7 μm) columns were obtained from Phenomenex (Torrance, CA, USA), while the Acclaim 120 C18 (2.1 × 150 mm, 5 μm) column was purchased from Thermo Fisher Scientific (Waltham, MA, USA). SPE cartridges, including Oasis HLB (500 mg, 6 mL and 1 g, 20 mL) and Oasis MAX (150 mg, 6 mL), were acquired from Waters Corporation (Milford, MA, USA). Bakerbond SPE cartridges—Silica (200 mg, 3 mL), Quaternary Amine (500 mg, 3 mL), C18 PolarPlus (500 mg, 6 mL), and SDB-1 (200 mg, 3 mL)—along with Bakerbond Speedisk C18, Speedisk C18 XF, and Speedisk H2O-Philic DVB disks were supplied by Avantor. Additionally, Bond Elut AccuCAT (200 mg, 6 mL), Bond Elut ENV (500 mg, 6 mL), and Bond Elut PPL (500 mg, 6 mL) SPE cartridges were procured from Agilent Technologies.

Wastewater samples were collected from one public hospital per voivodeship (16 hospitals in total) in Poland. These hospitals were selected to represent large cities with a high population density in relation to the voivodeship as a whole. The wastewater samples understood as “hospital outflows” were collected directly from the hospital premises through sewage wells in the period from February to March 2024, including sewage from all medical departments in a given hospital. The grab samples were collected at various depths (ranging from 1 to 14 m) depending on the hospital location and construction of the sewage system. These samples were immediately transported to the laboratory, centrifuged, filtered with 0.7 μm MN-GF-1 glass fiber filters purchased from MACHEREY-NAGEL (Düren, Germany), and subjected to SPE. Table 1 provides an overview of the voivodeship, including information on the number of inhabitants and population density in a given area, from which the samples were collected for the study. The statistical data were provided by Polish governmental organizations, including Statistics Poland and the Supreme Audit Office (NIK).

**Table 1:** Voivodeships and their characterization with regard to population and public hospitals.

No.	Voivodeship (province)	Voivodeship inhabitants (in thousands)^28^	Population density of voivodeship (per km^2^)^28^	No. of public hospitals in voivodeship^29^	No. of hospital beds in voivodeship^28^
1	Lower Silesian	2888.0	144.8	39	13.360
2	Kuyavian-Pomeranian	2006.9	111.7	28	8.399
3	Lublin	2024.6	80.6	36	10.406
4	Lubusz	980.0	70.1	17	3.863
5	Łódź	2378.5	130.5	31	11.160
6	Lesser Poland	3429.0	225.8	37	13.590
7	Masovian	5510.6	155.0	82	23.712
8	Opole	942.4	100.1	24	4.222
9	Subcarpathian	2079.1	116.5	28	8.702
10	Podlaskie	1143.4	56.6	24	5.293
11	Pomeranian	2358.3	128.6	25	7.820
12	Silesian	4346.7	352.4	69	21.652
13	Świętokrzyskie	1178.2	100.6	18	5.202
14	Warmian-Masurian	1366.4	56.5	33	6.336
15	Greater Poland	3493.6	117.1	49	13.629
16	West Pomeranian	1640.6	71.6	26	6.809

^28^Data from Statistics Poland in the year 2022;^29^Data from NIK on the day 30.06.2022.

According to the *Act on collective water supply and Collective Discharge of Waste Water* (Journal of Laws No. 72 of 2001, Pos. 747^30^ amended by Journal of Laws No. 85 of 2005 Pos. 728, 729^31^), the discharge of undisinfected waste water from hospitals into the sewerage system is not allowed in Poland. This applies especially to wastewater from isolation wards containing pathogenic microorganisms. There is no legal obligation to install WWTPs on hospital premises, and combined with the high cost of building them, only few hospitals have such treatment facilities. The most popular disinfection method is chlorination^32^, which alone shows effectiveness in removing some AMs from water, but these results should not be directly transferred to wastewater, which has different physicochemical properties and greater matrix complexity^33^.

### Preparation of standard solutions and quality control samples

Standard solutions of RAMs were prepared in concentrations of 1 mg mL^− 1^. FOF, LZD, CFD, CIL, and VBR standards were produced by dissolving the appropriate quantity of the substance in MeOH, while TGC was prepared with 0.1% FA in MeOH and ATM in a MeOH: H_2_O (80:20, V/V) mixture. The MEM standard was prepared using MOPS stabilizer solution according to the method described by Zou et al.^34^ Standard stock solutions were stored in the freezer at −20 °C.

Quality control (QC) samples for validation were prepared at three concentration levels in the matrix: high (HQC; 400 ng L^− 1^), medium (MQC; 250 ng L^− 1^), and low (LQC; 10 ng L^− 1^). The LQC, MQC and HQC concentrations were chosen to represent the lower, middle and upper parts of the calibration curve range. To reduce the impact of matrix effect on further analyses, the matrix selected for the QC samples was municipal wastewater filtered through a fiberglass filter and subjected to the selected SPE procedure described in section Development of sample pretreatment procedure. To ensure that no analytes were present in the wastewater used for its preparation, LC-MS/MS analysis of matrix was performed to confirm the absence of the selected RAMs. QC samples were prepared by spiking the matrix with the RAM working solutions, which were also prepared in the matrix.

Calibration solutions (CSs) were prepared in 10 concentrations (0.5, 2, 5, 25, 75, 100, 150, 250, 350, and 500 ng L^− 1^) by spiking the matrix with an appropriate volume of the RAM working solutions. The matrix was prepared in the same manner as for the QC samples.

### Development of the LC-MS/MS method for determination of RAMs

The development of the LC-MS-MS method entailed two steps: (i) selection of the detector working parameters for the selected RAMs and the ion source and (ii) selection of the chromatographic conditions.

The detector utilized for the study was a hybrid triple quadrupole-linear ion trap QTRAP 4000 tandem mass spectrometer obtained from AB Sciex LLC (Framingham, MA, USA), operating in multiple reaction monitoring (MRM) mode. The working parameters were selected using dedicated Analyst 1.5.1 software and are presented in Table 2. For each compound, two MRM transitions were selected: the parent ion (Q1) and the fragmentation ion (Q3), which correspond to the qualitative and quantitative signals, respectively. Additional parameters for the compounds included declustering potential (DP), collision energy (CE), collision cell exit potential (CXP), and entrance potential (EP). The ion source parameters for operation in positive electrospray ionization (ESI(+)) mode were as follows: curtain gas pressure (CUR = 40 psi), nebulizing gas pressure (GS1 = 60 psi), drying gas pressure (GS2 = 50 psi), ion source temperature (TEM = 550 °C), and ion spray voltage (IS = 5500 V). For ESI(−) mode, the conditions were: CUR = 50 psi, GS1 = 60 psi, GS2 = 70 psi, TEM = 550 °C, and IS = − 4500 V. The collision gas was set to high for both ionization modes.

**Table 2:** MS/MS working parameters for the selected RAMs.

	Q1 (*m/z*)	Q3 (*m/z*)	DP (V)	CE (V)	CXP (V)	EP (V)
*Compound parameters – ESI(+)*						
ATM	436.2	313.2/356.0	46	21/15	16/22	7
LZD	338.1	296.1/195.0	91	27/35	18/16	
MEM	384.2	68.1/141.1	61	67/25	10/10	
TGC	586.5	569.2/513.2	191	29/37	24/26	
CFD	547.3	468.2/59.2	66	19/79	26/10	
*Compound parameters – ESI(-)*						
FOF	136.9	78.9/63.1	−30	−26/−18	−1/−1	-7
VBR	296.0	234.1/278.2	−65	−28/−20	−7/−7	
CIL	357.0	226.3/313.2	−60	−26/−22	−5/−13	

*DP* declustering potential, *CE* collision energy, *EP* entrance potential, *CXP* collision cell exit potential.

The selection of chromatographic conditions included stationary phases and mobile phase eluents for the operation of a Dionex HPLC system, obtained from Dionex Corporation (Sunnyvale, CA, USA). Chromatographic columns with different types of packing (C18 and F5 sorbents) and various dimensions, as listed in section Chemicals and materials, were tested. To create a gradient elution system, a series of experiments were conducted in which the following combinations of solvents were tested: 0.1% FA in H_2_O + ACN, 0.1% FA in H_2_O + MeOH, 0.1% FA in H_2_O + 0.1% FA in MeOH, 10 mM ammonium acetate pH = 3 in H_2_O + 10 mM ammonium acetate pH = 3 in MeOH, and 0.3% FA in H_2_O + MeOH. The selection of stationary and mobile phases was undertaken to obtain intense peaks for each of the selected RAMs. The injection volume and mobile phase flow were adjusted to the chosen column conditions.

Under the specifications of the selected ZORBAX RRHD Eclipse Plus C18 (2.1 × 50 mm, 1.8 μm) column, the flow rate was set at 0.3 mL min^− 1^, the injection volume at 5 µL, and the temperature at 30 °C. A gradient system was employed for the analytes that ionized in ESI(+), with a percentage change in the mobile phase content of 0.3% FA in H₂O (A) and MeOH (B) starting at 0.0 min, when the composition was 90% A and 10% B. By 1.0 min, the ratio had been changed to 60% A and 40% B, which remained constant until 2.0 min. The gradient then underwent a change, reaching 50% A and 50% B at 6.0 min and 30% A and 70% B at 7.0 min. From 7.1 min, system stabilization began with the initial mobile phase composition. A gradient system lasting 10 min was employed for RAMs analyzed in negative ionization mode. This gradient began at 0.0 min with a composition of 90% A and 10% B, and by 2.0 min had been changed to 30% A and 70% B. At 3.5 min, the composition of the mobile phase was changed to 0% A and 100% B, which was maintained until 4.5 min. From 4.6 min onwards, the system began to stabilize with a composition of 90% A and 10% B, which lasted a further 3 min.

### Development of sample pretreatment procedure for extraction of RAMs

Before LC-MS/MS analysis wastewater sample pretreatment is necessary. To develop a universal method for concentration, purification, and enrichment of wastewater samples and subsequent chromatographic analysis, 26 different extraction procedures described in Supplementary Table S1 were tested. The changes included using various types of extraction sorbents packed in two ways – cartridges (procedures 4–26) and disks (procedures 1–3). For procedures with cartridges, the most commonly used sorbents were Oasis HLB sorbents with masses of 500 mg^–1^ g consisting of lipophilic divinylbenzene and hydrophilic N-vinylpyrrolidone with particle size of 30 μm. Oasis MAX mixed-mode anion exchange cartridges were also used. Bakerbond silica, quaternary amine and C18, styrene-divinylbenzene SDB1 and Varian Bond Elut Accucat (strong cation and strong anion exchange), ENV (polystyrene-divinylbenzene copolymer with larger particle sizes of 125 μm) and PPL (proprietary modified styrene-divinylbenzene stable at extreme pH) were also tested. Three disk sorbents were selected for this study – Bakerbond Speedisk C18, DVB and C18 XF for extra contaminated samples. All these various cartridges and discs were tested to provide all options for compounds with different physicochemical properties. Two different conditioning methods – 20 mL MeOH, 20 mL 0.1 M HCl for disks and 6 mL MeOH, 6 mL 0.1 M HCl, 6 mL H_2_O for cartridges were tested. The following five mixtures of eluent solutions were used: (1) MeOH, (2) MeOH: H_2_O (1:1, V/V), (3) MeOH, ACN, (4) acetone: MeOH: NH_4_OH (50:50:5; V/V/V), and (5) MeOH, 0.1% FA in H_2_O. The appropriate pH of the sample was selected (2.5, 3.0, 4.0, or 7.0) using four different acids and bases to obtain the most favorable conditions for the extraction of all the analytes. Moreover, the effect of the addition of EDTA on the extraction efficiency was examined.

Initially, 1 L of distilled water was enriched in the selected RAMs at a concentration of 500 ng L^− 1^. The sample was then adjusted to the appropriate pH, and in the meantime the SPE cartridge or disc was conditioned sequentially with the solvents listed in Supplementary Table S1. Subsequently, the sorbent was dried for 30 min under pressure using a BAKER SPE 12G system from Avantor. The compounds were then eluted using the appropriate solvent. Prior to LC-MS/MS analysis, the extract was evaporated to 1 mL under a nitrogen stream using a MultiVap 10 automatic system purchased from LabTech (Sorisole, Italy) and filtered with a PES 0.45 μm syringe filter. Three replicates were conducted for each procedure.

The selected procedure with the highest recoveries for the RAMs involved adjusting the pH of 1 L of wastewater sample to 2.5 with concentrated HCl, conditioning Oasis HLB (500 mg, 6 mL) cartridges successively with 6 mL of MeOH, 6 mL of 0.1 M HCl, and 6 mL of H_2_O, followed finally by elution with 6 mL of MeOH and 6 mL of 0.1% FA in H_2_O. The extracts were evaporated to 1 mL, filtered with a syringe filter, and subjected to LC-MS/MS analysis. Each sample was prepared in triplicate, each preparation being analyzed three times.

### Method validation

To confirm the suitability of the developed SPE-LC-MS/MS procedure, it is necessary to determine validation parameters. The validation included the determination of the linearity, sensitivity (LOD and LOQ), precision, accuracy, matrix effect (ME), and recovery (R) for each compound to ensure suitability for the intended use. The linearity of the method for the compounds was determined to be in the range of 0.5, 2.0 or 5.0 to 500 ng L^− 1^. The number of CSs and their preparation is described in Section Preparation of standard solutions and quality control samples. Linearity was confirmed by obtaining the relevant coefficient of determination (R^2^) value, and the regression equations were utilized to calculate the concentrations of RAMs in real samples. Method sensitivity was determined as the limit of detection (LOD) and limit of quantification (LOQ), which were calculated from the signal-to-noise ratio (S/N), when the LOD is the concentration when the S/N is equal to 3 and the LOQ when the S/N is equal to 10. The precision and accuracy of the method were also determined by the enrichment of the matrix solution at three concentration levels (LQC, MQC, and HQC), as described in section Preparation of standard solutions and quality control samples. The accuracy of the method was defined as the relative error (RE), while the precision was determined by the coefficient of variation (CV). The ME was evaluated at the abovementioned three concentration levels by comparing the peak areas of the analytes in the matrix solution to the peak areas of stock solutions of RAMs diluted with MOPS solution at pH = 7. In order to evaluate the efficiency of the method, 1 L of the same wastewater as that used for the matrix preparation was spiked with an RAM stock solution at a concentration level corresponding to the LQC, MQC, or HQC. The pretreatment procedure consisted of SPE using the selected procedure described in Section Development of sample pretreatment procedure. Recovery was calculated by comparing the ratio of the peak area of the RAM spiked before the SPE process to the peak area of the analyte spiked in the matrix at the same concentration. To determine each of the validation parameters, three replicates were conducted, and LC-MS/MS analyses were performed in triplicate.

## Results and discussion

### Development of LC-MS/MS method for RAM determination

In the first step of this study, the mobile and stationary phases were selected to provide intense, good-shaped signals for all the compounds simultaneously. The use of the ZORBAX RRHD Eclipse Plus C18 column yielded satisfactory intensities for all analytes, with both MRM transitions being visible for each compound. The selected column was characterized by a small particle size (1.8 μm), and it could be used at the acidic pH of the mobile phase. The Poroshell 120 EC-C18 column exhibited comparable separation effectiveness, yet the peaks demonstrated reduced symmetry and peak intensity compared to those obtained with the ZORBAX RRHD Eclipse Plus C18. In addition to the abovementioned columns, other with C18 packing, including the ZORBAX Eclipse Plus C18, and the Acclaim 120 C18, were found to be unsuitable for the selected RAMs, as was the pentafluorophenyl Kinetex F5.

As is typically the case for the LC-MS combination, the preferred organic solvents for the mobile phase are ACN and MeOH. These solvents could be used for RAM determination alone, as a mixture of both of them, or with the addition of ionization enhancers. As in the organic phase, additives such as acids (FA, trifluoroacetic acid, acetic acid, or oxalic acid), ammonia, buffers (phosphate, acetate, or formic acid), and H_2_O: ACN mixtures are frequently used in the aqueous phase^19,35–38^. During the study, a series of experiments were conducted to select the most effective solvent combinations for the mobile phase for the determination of RAMs. These included the use of H₂O and ACN, as well as H₂O and MeOH, with the addition of FA (either at a concentration of 0.1% or 0.3%), or else 10 mM ammonium acetate (pH = 3), both in water and organic solvents. From the above possibilities, 0.3% FA in H_2_O and MeOH were selected as the mobile phase components. ACN was rejected due to its excessive elution strength, which caused dead time elution for some analytes and split the peaks corresponding to MEM and TGC. The addition of FA, a typical ionization enhancer for ESI-MS, at a concentration of 0.1%, allowed well-separated peaks to be obtained for each compound. FA at a concentration of 0.3% significantly increased the signal intensity. The improved method’s sensitivity was linked to enhanced ionization efficiency and a lowering of the pH from 2.9 to 2.5, which was also the most favorable pH during the extraction. Using acetate buffer as part of the mobile phase additive resulted in mostly lower signal intensities, weaker peak separation, broadening, and splitting. Simultaneous addition of FA to the water and organic solvent did not result in any improvement.

**Fig. 1:**
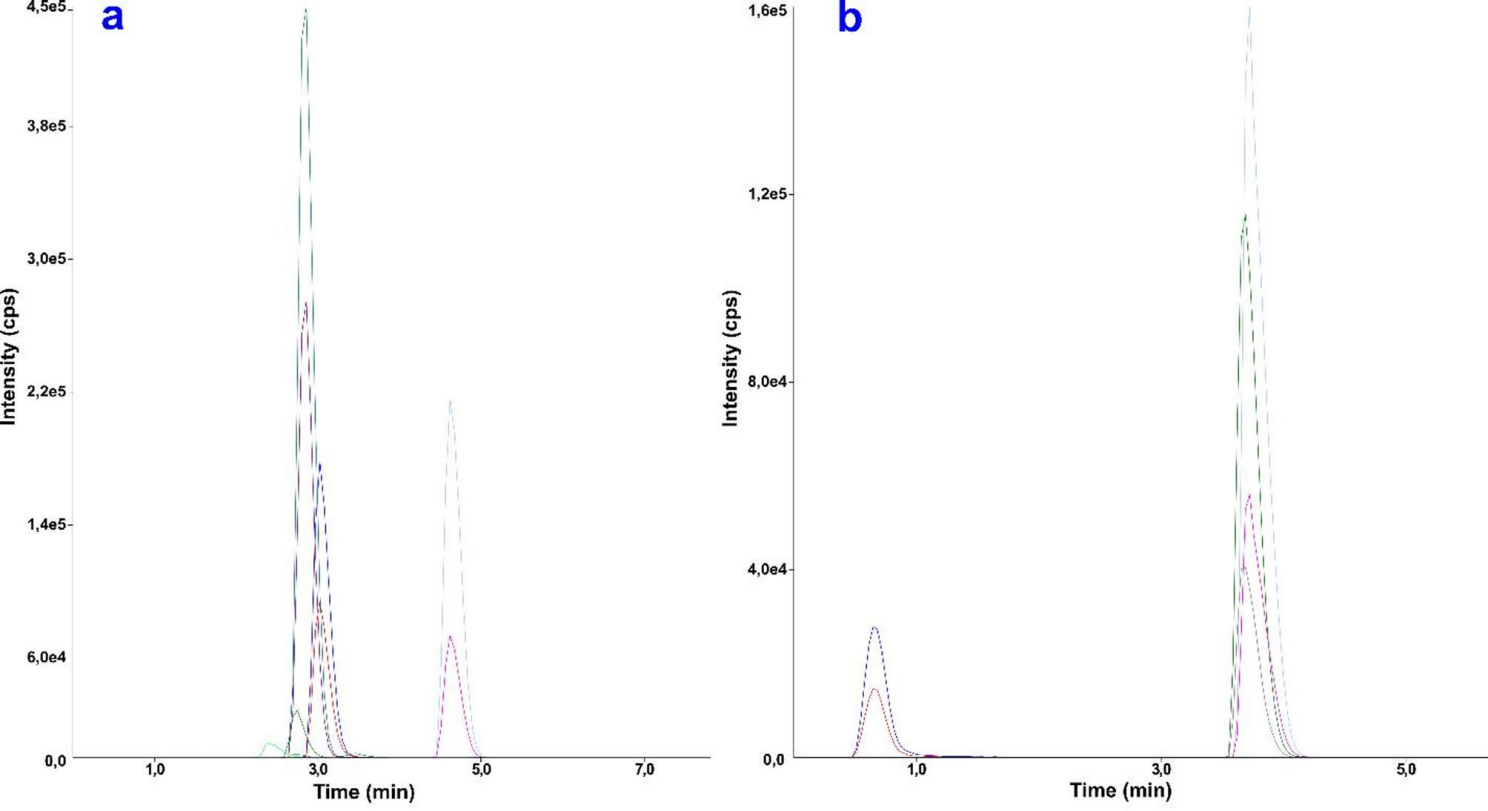
Chromatograms of RAM standard solution in concentration 500 ng mL^− 1^ each obtained for **a** ESI(+) and **b** ESI(-) methods.

Figure 1 shows the chromatogram of standard solutions of the RAMs selected for the study in positive (a) and negative (b) ionization.

### Development of sample pretreatment procedure

**Fig. 2:**
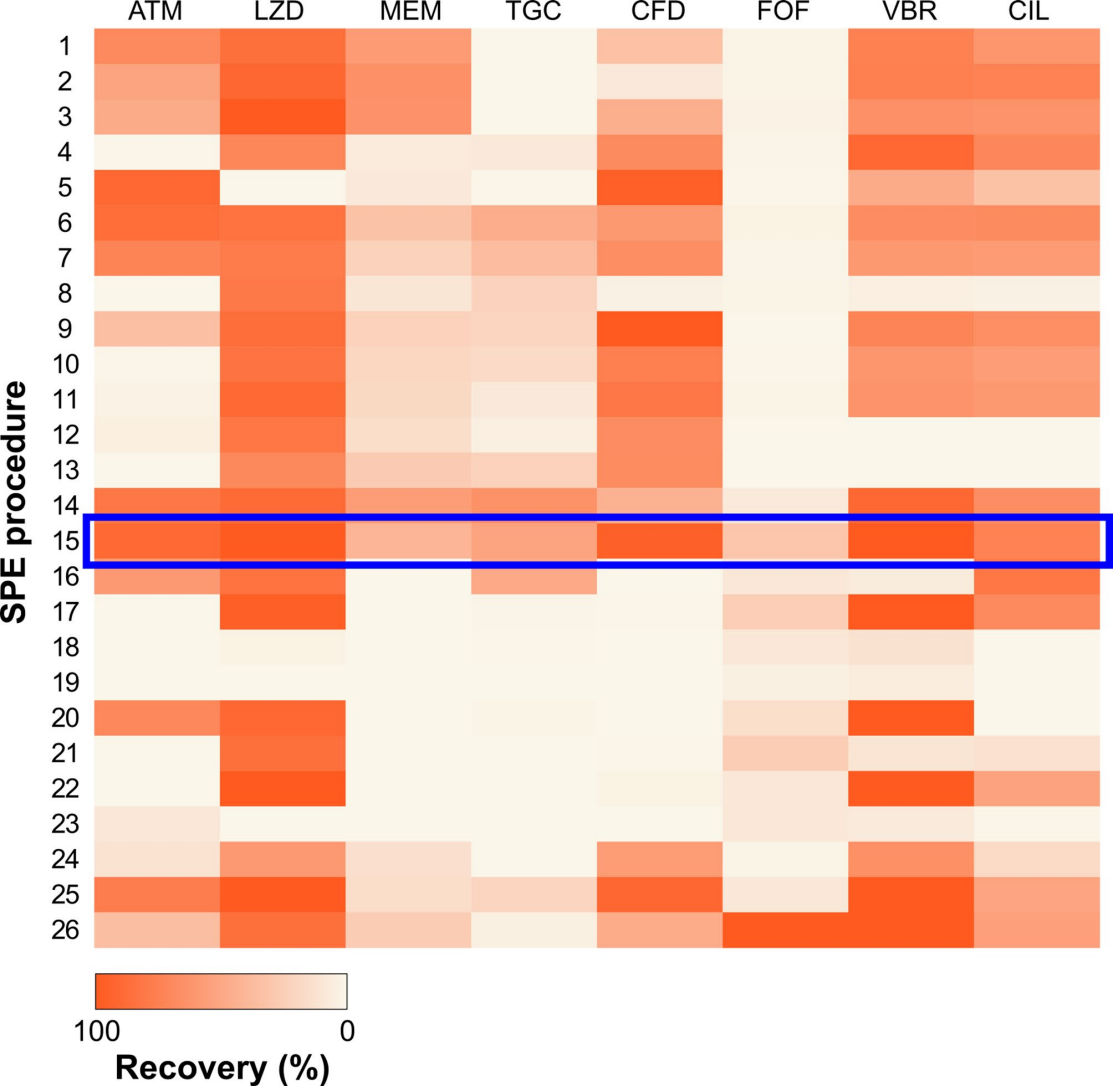
Heat map illustrating the recoveries obtained for selected RAMs during parameter selection for SPE (procedure selected for the determination of selected compounds in hospital wastewater is marked).

The treatment of environmental samples prior to LC-MS/MS analysis is always challenging due to the varied characteristics of analytes and the influence of a complex matrix. Moreover, meticulous development of each SPE parameter is key to achieving adequate extraction efficiency. During the development of the SPE procedure, the influence of the pH of the sample, the type of sorbent, its mass, eluents, and the addition of a chelating agent on the efficiency of the method were evaluated. After testing 26 procedures, the one that provided the highest recoveries for the analytes (procedure 15, Supplementary Table S1) was selected. The complexity of the environmental samples for which the method will be used, and the different compound characteristics of RAMs were considered. Based on the relatively high concentrations of RAMs in hospital wastewater previously described in the literature^19^, and taking into account the sensitivity and reproducibility of the obtained SPE-LC-MS/MS method, recoveries of at least 30% were accepted as sufficient. The chosen SPE procedure was characterized by simplicity and the use of basic, low-cost chemical reagents and the common Oasis HLB cartridge. It is also the first published extraction procedure that allows the simultaneous determination in environmental liquid samples of multiple AMs from the Reserve group, which are characterized by diversity in terms of size, mass, and the polarity of the molecule. The subsequent section of the article presents the concentrations of determined pharmaceuticals, which confirm the effectiveness of the developed method. Supplementary Table S2 provides recoveries obtained for all the tested extraction procedures. Figure 2 provides a visual comparison of RAM recoveries for all the procedures tested.

Prior to SPE, the samples were adjusted to a pH that was expected to cause ionization of the compounds and promote sorbent-analyte interactions that were appropriate for the extraction conditions. Four pH values (2.5, 3.0, 4.0 and 7.0) were tested to ensure maximum recovery of the target analytes, especially since the theoretical pK_a_ value of certain RAM can vary depending on the calculation method used^19^. For the selected RAMs, the optimal pH was found to be 2.5, which is consistent with the fact that the theoretically obtained pK_a_ for most of these compounds is above this value. This was particularly evident when comparing procedures 6 and 7. A change in pH from 3 to 2.5 resulted in an increase in R of 5% or more for most of analytes. Furthermore, the addition of EDTA to the sample was examined in procedures 9 and 14. The objective of the addition of chelating agents to liquid samples is to bind metals that can form complexes with some AMs or to prevent substances from sorbing onto glassware^39^. In the case of developing the method using distilled water, it was decided to check the effect of EDTA alone on the extraction efficiency of the RAMs. A comparison of procedure 14 and the finally selected 15 revealed a slight increase in R for MEM and TGC. This did not compensate for the reduction in recoveries for the other analytes (by as much as 50% in the case of CFD), so it was decided to eliminate the addition of EDTA when analyzing wastewater samples.

The highest recoveries were obtained for Oasis HLB (500 mg, 6 mL) cartridges. This sorbent, which exhibits hydrophilic and lipophilic properties, employs a mixed mode of retention, allowing for the simultaneous extraction of compounds of varying polarity from diverse samples due to the presence of hydrophilic N-vinylpyrrolidone and lipophilic divinylbenzene groups. Additionally, the sorbent demonstrated stability in acidic samples, which is a further advantage^40^. The 500 mg Oasis HLB was selected for 1 L samples due to its superior performance compared to the 1000 mg Oasis HLB (R under 70.4% for all analytes). Some of the other tested sorbents (procedures 17–26) demonstrated remarkably high recoveries for individual RAMs, which can be attributed to the optimal physicochemical properties of the sorbents for these AMs. Increased recoveries for some of the analytes were obtained with the Varian Bond Elut ENV and Varian Bond Elut PPL cartridges. However, these results were not sufficient to select these sorbents, as the versatile Oasis HLB sorbent was more suitable. The Varian Bond Elut AccuCAT cartridges were not suitable for any of the RAMs (*R* < 10%), despite their mixed-mode properties. However, it should be noted that these cartridges are designed for biological samples, which typically have smaller volumes than 1 L and have different matrix properties than those found in environmental samples^41^.

In order to improve the speed of extraction, three SPE sorbents in the form of disks (procedures 1–3) were tested. To the best of our knowledge, there is only one article that describes the use of disks to extract RAMs from environmental samples^42^. However, only two of the selected compounds were described. This sorbent packaging option allows for an increase in the flow rate and a significant reduction in the extraction time. The utilization of these sorbents resulted in comparable recoveries for all three sorbents, which were satisfactory for some analytes (*R* > 60% LZD, VBR, CIL, ATM for procedure 1, and MEM for procedures 2 and 3), but in most cases gave lower recoveries than those achieved with the selected procedure 15, particularly for ATM, TGC, CFD, FOF, and VBR. For this reason, it was decided not to use the disks for extraction purposes.

The conditioning of disks and cartridges varies due to the differing methods of packing the sorbent and the contact area with the sample. Consequently, larger volumes of solvents (20 mL) were used for disks. As conditioning solvents for the cartridges, 6 mL of MeOH, 6 mL of 0.1 M HCl, and 6 mL of H_2_O were used in sequence. These solvents had already been successfully employed by the authors in the development of a procedure for the extraction of AMs from environmental water samples^43^.

In the developed procedure, sequential elution using MeOH and then 0.1% FA in H₂O was chosen. The choice of such solvents allowed the simultaneous elution of RAMs that are soluble in MeOH and those that require an acidified or aqueous environment (Section Preparation of standard solutions and quality control samples). This resulted in significantly higher R values, particularly up to 30% higher for CFD than with elution using MeOH alone (procedure 6). The use of an acetone: MeOH: NH_4_OH mixture (50:50:5; V/V/V) for elution in procedure 16 resulted in lower recoveries, especially for VBR (R decrease of more than 90%). This result further suggests that the addition of H_2_O and an acidic environment are essential for adequate extraction efficiency of RAMs.

Table 3 compares the developed method with others that have been used to determine selected analytes in wastewater. Compared to other methods, which included a maximum of two selected analytes, the proposed method enables the simultaneous determination of the widest selection of RAMs (eight RAMs). Most methods included SPE extraction as a pretreatment step and LC-MS/MS as the analytical equipment for RAMs quantification. In our case, using the popular Oasis HLB cartridge was sufficient to determine eight compounds. Readily available solvents, such as methanol and acidified water, were used for elution. This supports the conclusion that our method is easily reproducible. During validation, our method produced greater sensitivity, with LOD and LOQ of 0.04 and 0.14 ng L^− 1^, respectively, whereas literature methods indicated higher values. The calibration curve range, which includes concentrations as low as 0.5 ng L^− 1^, is suitable for the studied samples, given the determined concentrations (Table 5). Most of the methods presented in the literature comparison do not allow quantification at such low concentrations.

**Table 3:** A comparison between the method developed during this study and the literature-reported methods for determining selected RAMs in wastewater.

Analysis method	Analyte	Wastewater type	Sample preparation technique	Analysis time (min)	1) LOD 2) LOQ (ng L^− 1^)	Linear range (ng L^− 1^)	Mean recovery (%)	Reference
LC-MS/MS	ATM LZD MEM TGC CFD FOF VBR CIL	Hospital wastewater	SPE Oasis HLB (6 mL, 500 mg) Elution: 6 mL MeOH, 6 mL 0.1% FA in H_2_O	15 (ESI+) 10 (ESI-)	1) 0.04–0.91 2) 0.14–3.03	0.5–500	31.49–103.69	This study
LC-MS/MS	MEM	Wastewater from swine farm	SPE Bond Elut PPL (3 mL, 200 mg) Elution: 4 mL 70% ACN	5 (+ stabilisation)	1) 50 2) 100	100–500 000	92.8	^44^
LC-MS/MS	MEM	Clinic-urban and rural wastewater	Sample dilution (1:1) with H_2_O: ACN (95:5, v/v) + 0.8 g L ^− 1^ EDTA	20	1) 33 2) 50	10–5 000	110	^20^
LC-MS/MS	MEM	Hospital wastewater	Tandem-SPE LC-NH_2_ (500 mg) Oasis HLB (500 mg) Elution: 9 mL MeOH	ni	1) 15.1 (MDL) 2) ni	ni	57	^45^
LC-UV	CFD	WWTP, influent, effluent	SPE Oasis HLB (6 mL, 200 mg) Elution: 6 mL MeOH	15	1) 11 200 (LOD) 33 (MDL) 2) 32 200	20–5 000	61.5	^46^
LC-MS/MS	CFD	WWTP influent, effluent	SPE Strata-X-A (6 mL, 500 mg) Elution: 10 mL 5% FA in MeOH	13	1) 9.1 2) ni	0.1–2 000	94.01	^47^
LC-MS/MS	LZD	WWTP influent, effluent	SPE Oasis HLB (3 mL, 60 mg) Elution: 6 mL 0.2% FA in ACN, 3 mL MeOH: Ace (1:1, v/v)	ni	1) 0.0217 2) ni	ni	ni	^27^
LC-MS/MS	LZD	WWTP influent, effluent	SPE Oasis HLB (60 mg) Elution: 5 mL MeOH: Ace (1:1, v/v), 3 mL 0.1% FA in MeOH	12	1) 0.67 2) ni	0.01–200	99.8	^48^
LC-MS/MS	MEM CFD	WWTP influent, effluent	Tandem SPE Chromabond SB (6 mL, 500 mg), Chromabond HR-X (6 mL, 500 mg) Elution: SB: 5 mL MeOH, 5 mL MeOH: Ace (50: 50, v/v), HR-X: 10 mL solvent for SB	25	1)2.5 25 (MDL) 2) 10 75 (MQL)	0.01–1000	47.0–65.3	^24,49^
LC-DAD/MS	CFD	Hospital wastewater WWTP influent, effluent	SPE Oasis HLB (6 mL, 500 mg) Elution: 10 mL MeOH	ni	ni	ni	ni	^21,50^
LC-MS/MS	MEM	Hospital wastewater	SPE Oasis HLB (500 mg) Elution: 5 mL MeOH, 5 mL of MeOH + 0.1 M acetic acid	18	ni	100–300 000	ni	^22^
LC-MS/MS	LZD	WWTP influent, effluent	SPE (in-house cartridge) (Oasis HLB 200 mg, Isolute ENV+ 150 mg, Strata-X-AW 100 mg, Strata-X-CV 100 mg) Elution: 4 mL of MeOH: ethyl acetate (1:1, v/v) with 2% am- monia, 2 mL MeOH: ethyl acetate (1:1, v/v) with 1.7% FA	20	ni	500–200 000	ni	^51^

*Ace* acetone, *ACN* acetonitrile, *ATM* aztreonam, *CFD* ceftazidime, *CIL* cilastatin, *DAD* diode array detector, *FA* formic acid, *FOF* fosfomycin, *LC* liquid chromatography, *LOD* limit of detection, *LOQ* limit of quantification, *LZD* linezolid, *MDL* method detection limit, *MEM* meropenem, *MeOH* methanol, *MQL* method quantification limit, *MS* mass spectrometry, *MS/MS* tandem mass spectrometry, *ni* no information, *SPE* solid-phase extraction, *TGC* tigecycline, *VBR* vaborbactam, *WWTP* wastewater treatment plan.

### Method validation

To verify the method’s sensitivity and reliability, a validation was performed, assessing parameters like linearity, LOD, LOQ, accuracy, precision, ME, and R. The results are outlined in Table 4. The calibration curves demonstrated linearity across a range of concentrations, from 0.5 to 500.0 ng L^− 1^ for LZD, MEM, CFD, and CIL, and from 2.0 to 500.0 ng L^− 1^ for ATM, FOF and 5.0 to 500.0 ng L^− 1^ for VBR. Two linear curves were constructed for TGC, one for concentrations ranging from 2.0 to 100.0 ng L^− 1^ and the other for concentrations from 100 to 500.0 ng L^− 1^. The R² were within the range of 0.9903 to 0.9999, confirming the linearity within the given ranges. The LOD and LOQ for the RAMs were in the range of 0.04 to 0.91 ng L^− 1^ and 0.14 to 3.03 ng L^− 1^, respectively. The CV was less than 5% for all analytes, indicating good method precision. The accuracy calculated as RE was up to 16.70%, with the majority of values falling within the ± 10% range. Considering the challenging environmental matrix that is wastewater, this can be considered an acceptable level of accuracy. Ion suppression was observed for the following analytes: ATM, LZD, TGC, VBR, and CIL. Conversely, ion enhancement was reported for MEM, CFD, and FOF. All of these exhibited relatively low ME, with a maximum of -18.56% for the LQC of FOF, leading to the conclusion that, in most cases, the ME can be considered negligible. The selectivity of the method was achieved by using the MRM mode to identify peaks in the blank samples that corresponded to the retention times of the RAMs. No interference peaks were identified in the blank wastewater samples at the targeted retention times. The recoveries were between 72.9% and 108.6% for ATM, LZD, CFD, VBR, and CIL, confirming the satisfactory extraction efficiency at each concentration level. According to the results obtained during the development of the SPE procedure, lower R values were obtained for MEM, TGC, and FOF during validation. However, the determination of these compounds was possible due to the high drug concentrations present in hospital wastewater and the relatively low detection limits of the developed method. In cases where RAMs concentrations were above the upper limit of the calibration curve, real samples were appropriately diluted and reanalyzed.

**Table 4:** Validation parameters obtained for the developed SPE-LC-MS/MS method.

RAM	Linearity (ng L^− 1^)	*R* ^2^	t_*R*_ (min)	LOD LOQ (ng L^− 1^)	QC (ng L^− 1^)	CV (%)	RE (%)	ME (%)	*R* (SD) (%)
ATM	2.0–500	0.9943	2.99	0.46 1.54	10 250 400	1.46 0.67 1.72	−10.99 −6.78 −8.03	3.85 6.64 9.15	100.2 (3.6) 93.6 (6.4) 94.4 (2.4)
LZD	0.5–500	0.9953	4.44	0.04 0.14	10 250 400	1.50 0.82 0.05	−16.61 −9.49 9.78	15.75 8.45 6.73	79.0 (4.3) 104.3 (0.8) 108.6 (3.1)
MEM	0.5–500	0.9938	2.82	0.13 0.42	10 250 400	0.81 0.69 0.22	8.47 −13.34 −1.46	−7.29 −8.43 −1.80	50.7 (3.8) 52.8 (7.8) 53.9 (4.8)
TGC	2.0–100 100–500	0.9924 0.9937	2.33	0.58 1.92	10 250 400	0.90 5.72 2.16	−16.48 2.05 −9.98	17.56 14.16 10.21	52.2 (8.6) 44.9 (1.1) 46.8 (4.7)
CFD	0.5–500	0.9954	2.71	0.04 0.15	10 250 400	1.02 1.57 0.69	11.91 1.47 2.26	−15.88 −11.96 −5.68	72.9 (7.9) 99.3 (3.8) 98.6 (6.4)
FOF	2.0–500	0.9903	0.68	0.41 1.35	10 250 400	1.32 0.92 0.78	−13.15 16.70 −7.79	−18.56 −8.23 −3.80	32.3 (8.2) 45.0 (8.0) 44.2 (8.9)
VBR	5.0–500	0.9993	3.46	0.91 3.03	10 250 400	1.34 1.16 1.81	−6.68 −4.05 13.12	10.39 5.93 7.64	97.9 (4.8) 82.1 (6.2) 86.6 (6.2)
CIL	0.5–500	0.9999	3.50	0.14 0.43	10 250 400	1.08 0.20 0.62	−4.49 9.70 −3.42	11.62 5.17 3.72	77.8 (6.4) 95.7 (6.3) 93.9 (4.5)

*RAM* Reserve antimicrobial, R^2^ coefficient of determination, t_R_ retention time, *LOD* limit of detection (S/*N* = 3), *LOQ * limit of quantification (S/*N* = 10), *QC* quality control, *CV* coefficient of variation, *RE* relative error, *ME* matrix effect, *R* recovery, *SD* standard deviation, *ATM* aztreonam, *LZD* linezolid, *MEM* meropenem, *TGC* tigecycline, *CFD* ceftazidime, *FOF* fosfomycin, *VBR* vaborbactam, *CIL* cilastatin.

### Determination of RAM concentrations in hospital wastewater samples

The selected eight RAMs were determined in 16 hospital wastewater samples. Table 5 lists the concentrations and standard deviations of the determined antibiotics for each hospital. The numbering of hospitals is consistent with the numbering of voivodeships in Table 1.

**Table 5:** Concentrations and standard deviations of quantified RAMs in hospital wastewater samples.

RAM	Hospital															
	1	2	3	4	5	6	7	8	9	10	11	12	13	14	15	16
	Concentration (SD) in ng L^− 1^															
ATM	4.45 (0.50)	37.19 (3.70)	27.62 (1.62)	8.25 (0.81)	25.76 (1.52)	nd	nd	4.84 (0.55)	27.93 (2.23)	nd	nd	nd	29.97 (2.92)	nd	nd	nd
LZD	22,488.8 (2,440.7)	4.34 (0.90)	215.51 (10.59)	< LOQ	4.71 (0.31)	2.50 (0.27)	146.43 (11.62)	5.10 (0.41)	144.50 (9.68)	nd	nd	2.16 (0.18)	165.13 (15.31)	12.82 (0.82)	7.74 (1.05)	1,364.6 (34.5)
MEM	nd	nd	nd	nd	nd	nd	23.08 (1.15)	nd	102.67 (8.91)	nd	nd	163.23 (14.64)	5.06 (0.37)	13.86 (0.42)	nd	nd
TGC	nd	nd	nd	nd	nd	nd	nd	nd	nd	nd	nd	nd	nd	nd	nd	nd
CFD	3,125.70 (174.0)	1.89 (0.98)	303.10 (13.32)	nd	nd	207.27 (15.96)	nd	nd	845.67 (55.53)	27.38 (1.76)	nd	27.47 (2.12)	nd	4,387.8 (357.9)	nd	nd
FOF	nd	nd	72.71 (8.05)	nd	nd	nd	nd	nd	nd	nd	nd	56.56 (7.91)	nd	nd	nd	nd
VBR	26.60 (2.11)	13.15 (0.73)	42.11 (3.51)	85.13 (3.93)	62.75 (4.35)	93.00 (2.57)	51.42 (2.88)	43.54 (1.86)	37.05 (2.72)	52.66 (4.07)	25.75 (1.56)	21.08 (0.62)	38.45 (2.07)	nd	59.29 (2.41)	35.51 (1.40)
CIL	6.00 (0.40)	3.12 (0.27)	16.67 (0.82)	26.74 (1.80)	10.90 (1.11)	nd	110.37 (4.37)	11.98 (0.87)	10.84 (0.28)	nd	11.41 (0.85)	5.11 (0.30)	5.89 (0.50)	nd	10.04 (0.39)	9.69 (0.75)

*RAM* Reserve antimicrobial, *ATM* aztreonam, *LZD* linezolid, *MEM* meropenem, *TGC* tigecycline, *CFD* ceftazidime, *FOF* fosfomycin, *VBR* vaborbactam, *CIL* cilastatin, *LOQ* limit of quantification (LZD = 0.14 ng L^− 1^), *nd* not detected.

According to Statistics Poland data, as of December 2023, Poland had a population of 37,635 thousand people in an area of 313,900 km². The country is divided into sixteen voivodeships, which are characterized by varying number of inhabitants and population density (Table 1), which tend to be higher in urban areas. The health care system in Poland is based on a system of public health insurance; most inpatient care is provided in hospitals, which are mostly public^52^. The number of hospitals increases with voivodeships inhabitants and population density, although there is not a clear correlation. The number of hospitals and hospital bed capacity should be analyzed at the local level, rather than correlated directly with population density at the voivodeship level. A similar situation is observed with the number and concentration of RAMs. In Hospital 9 of Subcarpathian voivodship, which is characterized by average population density and number of hospitals in relation to other voivodships, the highest number of RAMs was determined in the concentration range 10.84–845.67 ng L^− 1^ (6 out of 8 selected). The Silesian voivodeship has the highest population density and the second highest number of inhabitants. In Hospital 12 also 6 RAMs were determined in the concentration range 2.16–163.23 ng L^− 1^. Although the results are similar, these voivodships differ in demographics and number of hospitals. It is important to note here that the utilization of RAMs that are the focus of this study is limited exclusively to hospitals and other medical facilities. The lowest number of RAMs was observed in Hospital 10 from the Podlaskie and 11 from Pomeranian voivodeship. The voivodeships exhibit different demographic profiles, with varying numbers of inhabitants and population density, however, these values are relatively low to medium in comparison to the rest of the voivodeships. The highest concentrations of RAMs (> 1 µg L^− 1^) were recorded in Hospitals 1, 14 and 16, with LZD and CFD exceeding this limit in Hospital 1. The Lower Silesian voivodeship with Hospital 1 is the fifth in terms of population and the fourth in terms of population density. The highest number of inhabitants has Masovian voivodeship with Hospital 7, population density of 155 inhabitants per km^2^. One is not associated with a higher number of RAMs detected or higher concentrations of drugs determined. Again, there was no correlation between the increased population and the number of public hospital beds per voivodeship and the results obtained for each hospital. The above results indicate that the results for each hospital should be considered separately, without association with voivodeship-wide demographics. It is planned to extend the analysis to more hospitals per province.

The most prevalent RAM in wastewater from Polish hospitals was VBR. It was determined in samples from 15 out of 16 selected hospitals at concentrations of 13.15–93.00 ng L^− 1^. VBR is a representative of β-lactamase inhibitors, a group of compounds used to extend the stability of β-lactam antibiotics in the presence of carbapenemases produced as a defense by Gram-negative bacteria^53^. VBR is not used on its own, but is registered in combination with MEM and is classified as an RAM in this form on the AWaRe list^54^. Consequently, it is reasonable to infer that the presence of VBR in the wastewater indicates that MEM was also administered, which further compensates for the low R obtained for MEM. However, due to its instability in solution, which is typical for β-lactams^55^, it may have degraded earlier and be undetectable. MEM was quantified at four hospitals, with concentrations ranging from 5.06 to 163.23 ng L^− 1^. Despite the presence of MEM, no VBR was detected in wastewater from Hospital 14. This indicates that MEM may have been administered separately, as it is approved for use as a single drug^56^.

Similar to MEM, imipenem is very unstable in solution form. The presence of CIL in the wastewater indicates that it was administered along with imipenem. CIL is an inhibitor used to prevent imipenem hydrolysis caused by the human enzyme renal dehydropeptidase and bacterial metallo-β-lactamase^57^. It was quantified in 13 out of 16 hospitals at concentrations of 3.12–110.37 ng L^− 1^. Only the product with a composition of imipenem-CIL-relebactam is considered an RAM. Without relebactam, which was not determined, the other two drugs fall into the AWaRe Watch category, which is the group of important AMs with less restricted use and lower risk of AMR development^7^. CFD, like MEM, is an RAM only when used in combination with an inhibitor of bacterial enzymes that causes antibiotic degradation, but it can also be used alone. It has been detected in hospital wastewater at concentrations ranging from 1.89 ng L^− 1^ to 4.39 µg L^− 1^. It is one of the most commonly used AMs for nosocomial infections in critically ill patients^58^. The daily dosage of intravenously administered CFD can reach up to 6 g per day in adults^59^. Consequently, it is probable that the compound will be present in high concentrations in wastewater.

Another antibiotic frequently detected in hospital wastewater (14 out of 16 hospitals) was LZD, with a very wide range of concentrations (2.16 ng L^− 1^ to 22.49 µg L^− 1^). LZD is indicated for the treatment of skin infections and pneumonia. However, due to its activity against MDR Gram-positive bacteria, maximal oral bioavailability, and large tissue distribution, doctors requiring quick results often prescribe it off-label. However, its out of indication use is believed to pose a higher risk of adverse side effects^60^. Given this, it is reasonable to suspect that hospitals whose wastewater contained higher concentrations of LZD may have extensive dermatology or pulmonary treatment units.

ATM is an alternative for documented allergies to AMs from the penicillin group, which include β-lactam antibiotics^61^ (such as the CFD and MEM described in this paper). It is very important to use allergy screening tools to avoid unnecessary use of ATM and to preserve its therapeutic efficacy for as long as possible. In wastewater samples from Polish hospitals, it was determined at concentrations in the range of 4.45–37.19 ng L^− 1^ in half of the hospitals selected for the study. These are relatively low concentrations compared to other RAMs, which may suggest the implementation of proper antimicrobial stewardship in the use of ATM. FOF is a small polar molecule that may not be retained on the SPE sorbent. This, in conjunction with the low recoveries for this antibiotic, can make it challenging to detect and determine in real samples despite its widespread use. However, it is notable that FOF is excreted in a high percentage as a parent drug, which somewhat compensates for this defect. Furthermore, recommendations for the use of FOF indicate the administration of high oral doses of the drug, up to 3 g at a time or in divided doses that total 3 g daily. Intravenous dosing also includes high doses such as 6 g every 8 h for 7 days for urinary tract infections in adults^62^. This increases the likelihood of detecting the drug, despite the relatively poor extraction efficiency. TGC was not identified in any of the samples. It represents a novel class of tetracycline derivatives, the glycylcyclines, which are intended for use in complex infections when other antibiotics are deemed inappropriate^63^. Such recommendations, coupled with a substantial number of common and highly prevalent side effects, may result in the replacement of TGC by other antibiotics. Furthermore, the dosage of TGC (100 mg initially and then 50 mg every 12 h)^63^ is significantly lower than that of antibiotics such as FOF, which, in addition to a lower recovery rate, can make the determination of the drug more challenging.

As mentioned in the introduction, there are few reported works describing the determination of RAMs in hospital wastewater performed at this scale. Three different studies conducted in Singapore and Saudi Arabia describing the determination of MEM and CFD in hospital wastewater showed concentrations up to 1.07 µg L^− 1^.^22,45^ The situation was more serious in Romania, where the study included 3 hospitals and the highest CFD concentration was 10.46 µg L^− 1^, which is about 3 times higher than our results (maximum 3.13 µg L^− 1^).^21^ This indicates that, regardless of the continent, hospital outflows carry RAMs that are introduced into the environment in the absence of pretreatment.

The wide range of concentrations of RAMs detected, from a few ng to tens of µg per L, especially for LZD and CFD, indicates the variable use of these drugs in different hospitals. This is also related to their occurrence and distribution in the environment, especially since most of these drugs are excreted in unmetabolized form^19^. Hospital outflows are discharged into municipal wastewater due to the lack of pre-treatment processes in the facilities. As mentioned in the introduction, conventional WWTPs installed in Poland do not provide complete removal of pharmaceuticals during the treatment process, allowing them to enter water bodies in the first place, where they may be toxic to organisms living there. Even if AMs can be partially removed, there are still residues and even in concentrations of pg L^− 1^ may influence the induction of AMR and further its spread in both aquatic and terrestrial environments and subsequent transfer to humans^18,64^, posing a risk to public health and economy.

## Conclusions

The purpose of this study was to develop extraction and determination method for the selected RAMs and to determine these analytes in wastewater samples from 16 hospitals. Twenty-six procedures were tested for selection of SPE parameters such as sample pH and extraction additives, sorbent form and type, and elution solvents. The selected procedure proceeded as follows: 1 L of sample was adjusted to pH = 2.5, Oasis HLB sorbent (500 mg, 6 mL) was conditioned sequentially with 6 mL MeOH, 6 mL 0.1 M HCl, 6 mL H_2_O. After SPE, the sorbent was dried for 30 min and the analytes were eluted with two solvents: 6 mL MeOH and 6 mL 0.1% FA in H_2_O. The samples were evaporated to 1 mL under a gentle nitrogen stream, filtered and subjected to LC-MS/MS analysis, which was performed in two ESI modes: positive and negative for RAMs determination. The method was successfully validated, and the results ensure adequate robustness and reproducibility, showing high precision (CV < 5.72%) and acceptable accuracy (RE below 16.70%) over all QC concentrations. For most of the RAMs, the recoveries were very high (*R* > 75%), making this approach a suitable tool for future environmental monitoring. Another advantage was the good reproducibility of the sample pretreatment process. The low LOD and LOQ of the method provided high sensitivity, allowing reliable determination of selected RAMs in wastewater samples. Despite some challenges in validation, sample preconcentration 1000 times prior to analysis, along with the presence of high concentrations of RAMs in hospital outflows helped to mitigate the lower extraction recoveries observed for a few compounds. RAMs were detected in a very wide range of concentrations (from 1.89 ng L^− 1^ to 22.49 µg L^− 1^) in samples from the hospitals involved in the project. The occurrence of these substances in wastewater can be considered a clear indication of their presence in the environment, given the limited removal capacity of conventional WWTPs, which are typical for Poland and several other countries. Measuring the presence and concentrations of AMs in the environment is essential for evaluating their effects on animals and ecosystems and serves as a basis for subsequent ecotoxicological assessments. Some AMs can accumulate in the environment and potentially interfere with metabolic processes in plants. While antibiotics are generally considered to be persistent or pseudo-persistent, their degradation products may be more toxic than the parent compounds. Additionally, the persistence of AMs in the environment can contribute to the selection and spread of resistant bacterial strains. These resistance traits can be transferred to other bacteria that continue to cause infections in humans and animals^3,65^. However, data on these aspects remain limited for RAMs, highlighting the need for further investigation. This study contributes to filling that gap by providing practical tool for detecting selected RAMs in hospital wastewater. Wider application of the developed SPE-LC-MS/MS method may support efforts to monitor RAMs concentrations in other liquid environmental samples and contribute to a more comprehensive understanding of RAMs occurrence and distribution in the environment.

## Electronic supplementary material

Below is the link to the electronic supplementary material.

Supplementary Material 1

## Data Availability

All data generated and analyzed during our study are included in this article.
