# Supplementary material for: Development SPE-LC-MS/MS method for determination of WHO AWaRe Reserve antibiotics in hospital wastewater
Source: Sci Rep. 2025 Jul 1;15:22163. doi: 10.1038/s41598-025-04951-z (PMC12214922; doi:10.1038/s41598-025-04951-z)
Supplement: Supplementary file 1 — Supplementary Material 1 [file 41598_2025_4951_MOESM1_ESM.docx]

**Development SPE-LC-MS/MS method for determination of WHO AWaRe Reserve antibiotics in hospital wastewater**

Joanna Wilk^1^, Paulina Sowik^2^, Ewa Felis^2^, Monika Harnisz^3^, Ewa Korzeniewska^3^,
Sylwia Bajkacz^1,4^*

*^1^Department of Inorganic Chemistry, Analytical Chemistry, and Electrochemistry, Faculty of Chemistry, Silesian University of Technology, Krzywoustego 6 Str., 44-100, Gliwice, Poland*

*^2^ Department of Environmental Biotechnology, Faculty of Energy and Environmental Engineering, Silesian University of Technology, Akademicka 2 Str., 44-100, Gliwice, Poland*

*^3^Department of Engineering of Water Protection and Environmental Microbiology, Faculty of Geoengineering, University of Warmia and Mazury in Olsztyn, Prawocheńskiego 1 Str., 10-720, Olsztyn, Poland*

*^4^Biotechnology Centre, Silesian University of Technology, Krzywoustego 8 Str., 44-100, Gliwice, Poland*

*Corresponding author: Sylwia Bajkacz, e-mail: Sylwia.Bajkacz@polsl.pl

**SUPPLEMENTARY MATERIAL**

**Summary**

This supporting information file includes additional results and information as described in the text of the main article including:

Table S1. Parameters modified during SPE procedure development for selected RAMs

Table S2. Comparison of 26 SPE procedures in the case of obtained recovery (R)

Table S1. Parameters modified during SPE procedure development for selected RAMs

| **Procedure** | **Cartridge** | **Conditioning solvents** | **Sample pH (adjusting solvent)** | **Elution solvent** | **Additives** |
| --- | --- | --- | --- | --- | --- |
| **1** | Bakerbond Speedisk C18 | 20 mL MeOH, 20 mL 0.1 M HCl, 20 mL H_2_O | 2.5 (HCl) | 40 mL MeOH |  |
| **2** | Bakerbond Speedisk H_2_O Philic DVB | 20 mL MeOH, 20 mL 0.1 M HCl, 20 mL H_2_O | 2.5 (HCl) | 40 mL MeOH |  |
| **3** | Bakerbond Speedisk C18 XF | 20 mL MeOH, 20 mL 0.1 M HCl, 20 mL H_2_O | 2.5 (HCl) | 40 mL MeOH |  |
| **4** | Oasis HLB  (500 mg, 6 mL) | 6 mL MeOH,  6 mL 0.1 M HCl, 6 mL H_2_O | 4.0 (HCl) | 12 mL MeOH |  |
| **5** | Oasis HLB  (500 mg, 6 mL) | 6 mL MeOH,  6 mL 0.1 M HCl, 6 mL H_2_O | 4.0 (HCl) | 12 mL MeOH:H_2_O (1:1) |  |
| **6** | Oasis HLB  (500 mg, 6 mL) | 6 mL MeOH,  6 mL 0.1 M HCl, 6 mL H_2_O | 2.5 (HCl) | 12 mL MeOH |  |
| **7** | Oasis HLB  (500 mg, 6 mL) | 6 mL MeOH,  6 mL 0.1 M HCl, 6 mL H_2_O | 3.0 (HCl) | 12 mL MeOH |  |
| **8** | Oasis HLB  (500 mg, 6 mL) | 6 mL MeOH,  6 mL 0.1 M HCl, 6 mL H_2_O | 7.0 (NH_4_OH) | 12 mL MeOH |  |
| **9** | Oasis HLB  (500 mg, 6 mL) | 6 mL MeOH,  6 mL 0.1 M HCl, 6 mL H_2_O | 4.0 (HCl) | 12 mL MeOH | + 250 mg EDTA |
| **10** | Oasis HLB  (500 mg, 6 mL) | 6 mL MeOH,  6 mL 0.1 M HCl, 6 mL H_2_O | 4.0 (HCl) | 6 mL MeOH, 6 mL ACN |  |
| **11** | Oasis HLB  (500 mg, 6 mL) | 6 mL MeOH,  6 mL 0.1 M HCl, 6 mL H_2_O | 4.0 (0.1 M HCl) | 12 mL MeOH |  |
| **12** | Oasis HLB  (500 mg, 6 mL) | 6 mL MeOH,  6 mL 0.1 M HCl, 6 mL H_2_O | 4.0 (H_2_SO_4_) | 12 mL MeOH |  |
| **13** | Oasis HLB  (1 g, 20 mL) | 6 mL MeOH,  6 mL 0.1 M HCl, 6 mL H_2_O | 4.0 (HCl) | 12 mL MeOH |  |
| **14** | Oasis HLB  (500 mg, 6 mL) | 6 mL MeOH,  6 mL 0.1 M HCl, 6 mL H_2_O | 2.5 (HCl) | 12 mL MeOH | + 250 mg EDTA |
| **15** | Oasis HLB  (500 mg, 6 mL) | 6 mL MeOH,  6 mL 0.1 M HCl, 6 mL H_2_O | 2.5 (HCl) | 6 mL MeOH, 6 mL 0.1% FA in H_2_O |  |
| **16** | Oasis HLB  (500 mg, 6 mL) | 6 mL MeOH,  6 mL 0.1 M HCl, 6 mL H_2_O | 2.5 (HCl) | 12 mL acetone:MeOH:NH_4_OH (50:50:5; V/V/V) |  |
| **17** | Oasis MAX  (150 mg, 6 mL) | 6 mL MeOH,  6 mL 0.1 M HCl, 6 mL H_2_O | 2.5 (HCl) | 12 mL MeOH |  |
| **18** | Bakerbond Silica | 6 mL MeOH,  6 mL 0.1 M HCl, 6 mL H_2_O | 2.5 (HCl) | 12 mL MeOH |  |
| **19** | Bakerbond Quart Amine | 6 mL MeOH,  6 mL 0.1 M HCl, 6 mL H_2_O | 2.5 (HCl) | 12 mL MeOH |  |
| **20** | Bakerbond C18 Polar Plus | 6 mL MeOH,  6 mL 0.1 M HCl, 6 mL H_2_O | 2.5 (HCl) | 12 mL MeOH |  |
| **21** | Oasis MAX  (150 mg, 6 mL) | 6 mL MeOH,  6 mL 0.1 M HCl, 6 mL H_2_O | 2.5 (HCl) | 12 mL acetone:MeOH:NH_4_OH (50:50:5; V/V/V) |  |
| **22** | Oasis MAX  (150 mg, 6 mL) | 6 mL MeOH,  6 mL 0.1 M HCl, 6 mL H_2_O | 2.5 (HCl) | 6 mL MeOH,  6 mL 0.1% FA in H_2_O |  |
| **23** | Varian Bond Elut Accucat | 6 mL MeOH,  6 mL 0.1 M HCl, 6 mL H_2_O | 2.5 (HCl) | 6 mL MeOH,  6 mL 0.1% FA in H_2_O |  |
| **24** | Bakerbond SDB1 | 6 mL MeOH,  6 mL 0.1 M HCl, 6 mL H_2_O | 2.5 (HCl) | 6 mL MeOH,  6 mL 0.1% FA in H_2_O |  |
| **25** | Varian Bond Elut ENV | 6 mL MeOH,  6 mL 0.1 M HCl, 6 mL H_2_O | 2.5 (HCl) | 6 mL MeOH,  6 mL 0.1% FA in H_2_O |  |
| **26** | Varian Bond Elut PPL | 6 mL MeOH,  6 mL 0.1 M HCl, 6 mL H_2_O | 2.5 (HCl) | 6 mL MeOH,  6 mL 0.1% FA in H_2_O |  |

Table S2. Comparison of 26 SPE procedures in the case of obtained recovery (R)

| Procedure | R (%) | | | | | | | |
| --- | --- | --- | --- | --- | --- | --- | --- | --- |
|  | **ATM** | **LZD** | **MEM** | **TGC** | **CFD** | **FOF** | **VBR** | **CIL** |
| 1 | 70.04 | 86.18 | 58.86 | 0.40 | 34.38 | 2.38 | 74.89 | 62.12 |
| 2 | 53.33 | 91.70 | 65.24 | 0.00 | 8.74 | 1.91 | 76.29 | 74.63 |
| 3 | 48.53 | 102.08 | 64.28 | 0.00 | 45.99 | 2.79 | 65.46 | 64.00 |
| 4 | 0.83 | 71.91 | 7.13 | 8.77 | 69.08 | 1.32 | 91.04 | 71.39 |
| 5 | 91.29 | 0.17 | 8.52 | 1.10 | 96.71 | 1.11 | 48.26 | 33.84 |
| 6 | 88.06 | 84.39 | 32.76 | 46.28 | 60.25 | 2.99 | 67.48 | 69.00 |
| 7 | 73.62 | 79.36 | 22.80 | 37.45 | 66.90 | 1.34 | 60.16 | 58.73 |
| 8 | 0.17 | 80.28 | 10.43 | 22.75 | 3.21 | 1.63 | 4.90 | 3.23 |
| 9 | 35.29 | 87.38 | 23.27 | 21.62 | 128.95 | 0.51 | 72.91 | 66.66 |
| 10 | 1.14 | 83.45 | 20.16 | 17.42 | 76.60 | 0.92 | 61.73 | 57.37 |
| 11 | 2.71 | 90.14 | 19.76 | 8.79 | 80.84 | 2.15 | 63.66 | 60.37 |
| 12 | 5.03 | 81.71 | 15.88 | 4.58 | 68.02 | 0.00 | 0.00 | 0.00 |
| 13 | 0.58 | 70.37 | 27.93 | 23.40 | 68.46 | 0.00 | 0.00 | 0.00 |
| 14 | 81.23 | 89.55 | 57.38 | 64.37 | 44.03 | 8.27 | 90.89 | 66.80 |
| 15 | 90.22 | 99.97 | 41.97 | 52.98 | 96.42 | 31.49 | 103.69 | 74.57 |
| 16 | 59.76 | 84.61 | 0.25 | 49.37 | 0.24 | 9.49 | 7.01 | 82.01 |
| 17 | 0.02 | 97.26 | 0.04 | 1.43 | 1.03 | 25.76 | 118.84 | 69.77 |
| 18 | 0.16 | 3.13 | 0.14 | 0.73 | 0.02 | 9.58 | 13.31 | 0.47 |
| 19 | 0.11 | 0.15 | 0.06 | 0.58 | 0.04 | 4.18 | 6.03 | 0.34 |
| 20 | 71.13 | 91.21 | 0.01 | 1.57 | 0.02 | 15.38 | 108.21 | 0.05 |
| 21 | 0.03 | 86.58 | 0.11 | 0.00 | 0.68 | 26.43 | 11.22 | 13.58 |
| 22 | 0.04 | 101.66 | 0.17 | 0.00 | 3.17 | 10.01 | 123.90 | 54.42 |
| 23 | 9.97 | 0.54 | 0.00 | 0.00 | 0.00 | 9.46 | 7.80 | 1.10 |
| 24 | 12.77 | 59.73 | 14.77 | 0.00 | 58.28 | 2.36 | 65.96 | 18.05 |
| 25 | 77.63 | 118.59 | 16.47 | 21.66 | 91.79 | 8.99 | 136.30 | 52.65 |
| 26 | 36.02 | 86.52 | 27.18 | 4.38 | 47.56 | 248.94 | 115.03 | 55.81 |

ATM – aztreonam, LZD – linezolid, MEM – meropenem, TGC – tigecycline, CFD – ceftazidime, FOF – fosfomycin,
VBR – vaborbactam, CIL – cilastatin
